# Methanesulfonate (MSA) Catabolic Genes from Marine and Estuarine Bacteria

**DOI:** 10.1371/journal.pone.0125735

**Published:** 2015-05-15

**Authors:** Ana C. Henriques, Paolo De Marco

**Affiliations:** Instituto de Investigação e Formação Avançada em Ciências e Tecnologias da Saúde, CESPU, Rua Central de Gandra 1317, 4585–116 Paredes, Portugal; Universidad Miguel Hernandez, SPAIN

## Abstract

Quantitatively, methanesulfonate (MSA) is a very relevant compound in the global biogeochemical sulfur cycle. Its utilization by bacteria as a source of carbon and energy has been described and a specific enzyme, methanesulfonate monooxygenase (MSAMO), has been found to perform the first catabolic step of its oxidation. Other proteins seemingly involved in the import of MSA into bacterial cells have been reported. In this study, we obtained novel sequences of genes *msmA* and *msmE* from marine, estuary and soil MSA-degraders (encoding the large subunit of the MSAMO enzyme and the periplasmic component of the import system, respectively). We also obtained whole-genome sequences of two novel marine *Filomicrobium* strains, Y and W, and annotated two full *msm* operons in these genomes. Furthermore, *msmA* and *msmE* sequences were amplified from North Atlantic seawater and analyzed. Good conservation of the MsmA deduced protein sequence was observed in both cultured strains and metagenomic clones. A long spacer sequence in the Rieske-type [2Fe-2S] cluster-binding motif within MsmA was found to be conserved in all instances, supporting the hypothesis that this feature is specific to the large (α) subunit of the MSAMO enzyme. The *msmE* gene was more difficult to amplify, from both cultivated isolates and marine metagenomic DNA. However, 3 novel *msmE* sequences were obtained from isolated strains and one directly from seawater. With both genes, our results combined with previous metagenomic analyses seem to imply that moderate to high-GC strains are somehow favored during enrichment and isolation of MSA-utilizing bacteria, while the majority of *msm* genes obtained by cultivation-independent methods have low levels of GC%, which is a clear example of the misrepresentation of natural populations that culturing, more often than not, entails. Nevertheless, the data obtained in this work show that MSA-degrading bacteria are abundant in surface seawater, which suggests ecological relevance for this metabolic group of bacteria.

## Introduction

The ocean constitutes a large reservoir of sulfur and hence the transfer of volatile sulfur compounds from the sea to the atmosphere represents a key process in the sulfur cycle [1]. Dimethylsulfide (DMS) is the main component of marine emissions of volatile sulfur, contributing an estimated 98% of atmospheric DMS [2]. DMS is produced in the marine environment by degradation of its precursor dimethylsulfoniopropionate (DMSP) [3], which in turn is synthesized by microalgae and cyanobacteria starting from seawater sulfate. Only a small portion of the DMS produced in the oceans escapes to the atmosphere. Here it can be oxidized by the action of radical species such as OH, BrO, Cl and/or NO_3_, through several intermediate reactions, forming a variety of products such as sulfur dioxide (SO_2_), methanesulfonic acid (MSA) and dimethylsulfoxide (DMSO) [1,4,5].

MSA (formula CH_3_SO_3_H, the smallest organic sulfonic acid) is one of the main products of DMS oxidation since it is estimated that 25–70% of the flux of dimethylsulfide is oxidized to methanesulfonate (approximately 10^10^ Kg/year) [2,6–10]. Due to its hygroscopic nature, MSA takes part in the formation of cloud condensation nuclei (CCN), contributes to the regulation of cloud formation and thus has a significant impact on albedo regulation [4,11,12]. MSA falls onto lands and oceans in wet and dry precipitation [2,10,13] and, despite its high chemical stability, has only been found to accumulate in detectable levels in the frozen layers of snow of Antarctica and Greenland [14–16]. MSA can be used as a sulfur source by some aerobic bacteria [10]. On the other hand, several methylotrophic bacteria isolated from different environments have shown the ability to grow using MSA as the sole source of carbon and energy. The soil bacterium *Methylosulfonomonas* (*Me*.) *methylovora* strain M2 was the first such isolate to be described [17–19], followed by the marine *Marinosulfonomonas* (*Ma*.) *methylotropha* strains TR3 and PSCH4 [20,21]. All of these isolates contained an inducible multicomponent cytoplasmic enzyme, MSA monooxygenase (MSAMO). This enzyme is responsible for splitting the C-S bond, catalyzing the first oxidative step of MSA to the central methylotrophic intermediate formaldehyde with the release of sulfite, which is subsequently oxidized to sulfate. Formaldehyde was assimilated through the serine cycle or fully oxidized to CO_2_ and H_2_O, in order to yield reducing power and energy. MSAMO was purified from *Me*. *methylovora* str. M2 and its four components, all necessary for enzyme activity, were identified: the large (or α) subunit of the hydroxylase (MsmA—48 kDa), the small (or β) subunit of the hydroxylase (MsmB—20 kDa), a 16 kDa ferredoxin (MsmC), and a 38 kDa reductase component (MsmD) [10,18,22,23]. Subsequently, the genes encoding MSAMO (*msmA*, *msmB*, *msmC* and *msmD*) were cloned and sequenced from *Me*. *methylovora* str. M2 and *Ma*. *methylovora* str. TR3. In the case of *Me*. *methylovora* str. M2, an *msmABCD* operon was found [18]. The *msm* genes from *Ma*. *methylotropha* str. TR3 were located in two separate but complementary operons, *msmABC* and *msmABD* [24], which showed general sinteny and high similarity levels with the corresponding operon from *Me*. *methylovora* str. M2 [15,24].

Several other bacterial strains capable of growing on MSA as the sole carbon and energy source have since been isolated from soil (*Hyphomicrobium*, *Methylobacterium* [24,25]), river sediments (*Methylobacterium*, *Flavobacterium*, *Rhodococcus*, *Afipia felis* [18,26]), seawater (*Pedomicrobium* [24]) and Antarctic lakes (*Afipia felis* [26]. Most of them have been tested for the presence of the *msm* genes either by Southern blotting [25] or by PCR with a set of primers targeting the *msmA* gene [24,26].

In addition to the results obtained with cultured isolated strains, *msmA* sequences were also amplified by Baxter et al. [24] from DNA extracted directly from a soil sample and from soil and marine enrichments. A search for *msm* gene homologues in the Sargasso Sea Metagenome (SSM) database [27] was performed by Leitão et al. [15] who retrieved two scaffolds bearing genes with high identity to the *msmABCD* cluster and several singletons with high identity to shorter segments of the *msm* clusters or individual *msm* genes.

The predicted gene product of gene *msmA* revealed a unique sequence in the region associated to the Rieske-type [2Fe–2S] cluster, with a longer-than-usual 26-amino acid spacer between the two highly conserved cysteine—histidine groups in the **C**X**H**-X_n_-**C**XX**H** conserved motif [15,18,24,26]. However, not all the sequences annotated as MsmA in the databases include this motif. The genome of the first cultured representative of the marine SAR116 clade, *Candidatus Puniceispirillum marinum* str. IMCC1322, was sequenced [28] and two hypothetical proteins were annotated as hydroxylase alpha subunit of MSAMO (MsmA), but only one of these (NCBI Reference Sequence: YP_003552429) contains the **C**X**H**-X_n_-**C**XX**H** Rieske-associated motif. The absence of this element is observed in many predicted MsmA proteins in the Global Ocean Sampling (GOS) metagenomic data [29] and marine virome sequences, including the one found in bacteriophage HMO-2011, which is known to infect *C*. *Puniceispirillum marinum* str. IMCC1322 [30]. On the other hand, the predicted proteomes of methylotrophic strains *Methylibium petroleiphilum* str. PM1 [31] and *Methyloversatilis universalis* str. FAM5 [32] contain polypeptides annotated as MsmA with a shorter spacer (n = 17) in the Rieske-associated motif. However, all the organisms so far isolated for which growth on MSA has been positively proved revealed the presence of a Rieske motif with a longer spacer [18,24].

The genes involved in MSA transport have also been investigated. De Marco et al. [18] and Jamshad et al. [33] sequenced and analyzed the *msmEFGH* operon from *Me*. *methylovora* str. M2, adjacent to operon *msmABCD* but transcribed in the opposite direction. The protein components encoded by these genes were proposed to constitute an MSA/sulfonate transport system belonging to the ABC-type superfamily of transporters. *msmE* and *msmF* genes would encode, respectively, a putative periplasmic substrate-binding protein and a putative membrane-associated protein, while the products of *msmG* and *msmH* were proposed to be, respectively, an ATP-binding protein and an outer membrane-associated permease. The search for *msm* genes in the Sargasso Sea Metagenome by Leitão et al. [15] also yielded a scaffold bearing genes highly identical to the *msmEFGH* operon.

More recent evidence also suggests that the *msm* genes are functionally very active in the oceanic environment. In a metatranscriptome study [34] it was shown that in North Atlantic coastal seawater collected at Sapelo Island (Georgia, USA) *msm* genes similar to those found in *C*. *Puniceispirillum marinum* str. IMCC1322 were highly expressed.

In this work, we looked for *msmA* and *msmE* gene sequences in previously isolated MSA-degrading strains, as well as in the genomes of two novel marine *Filomicrobium* isolates. We also amplified and analyzed *msmA* and *msmE* gene sequences from coastal ocean surface water metagenomic DNA in order to extend our knowledge on these ecofunctional genetic markers of MSA degradation.

## Material and Methods

### Enrichment and isolation of marine strains Y and W

A 10 L ocean surface water sample was collected roughly 5 Km off the coast of Matosinhos, Portugal (approximate coordinates 41.1822, -8.7587), and biomass was obtained by successive filtration through 8 μm, 0.45 μm and 0.2 μm filters. Since sampling was performed in publicly accessible ocean waters and involved no environmental risk or damage and no commercial exploitation, no specific permissions were required. This study did not involve endangered or protected species. This biomass was then resuspended in the final 200 mL of the ocean water sample and incubated aerobically at room temperature (15 to 25°C) in the dark, continuously mixed by a magnetic stirrer. Two milliliters of alkaline (pH 8) sodium methanesulfonate 1 M were added to the suspension (10 mM final concentration) and similar amounts were used to spike the enrichment every time a drop to pH 6 or lower was observed. This schedule was maintained over 2 months. The enrichment began to become turbid with cells and a salt precipitate. Small samples were periodically removed to inoculate solid minimal medium MinE [35] amended with 3% NaCl and sodium methanesulfonate 10 mM. Several types of bacterial colonies grew on the agar plates, but most did not survive replication. Two strains grew well in these conditions, one producing white colonies (strain W) and one presenting a yellow pigmentation (strain Y). Amplification and sequencing of the SSU rRNA gene from the two strains revealed that both belonged to the genus *Filomicrobium* within the *Hyphomicrobiaceae* (*Alphaproteobacteria)* and had almost identical 16S rRNA gene sequences (99.7% identity).

### DNA extraction from isolated MSA-degrading bacteria

Previously described strains used in this work were: *Methylobacterium* sp. str. P1 and *Hyphomicrobium* sp. str. P2 [25], *Methylobacterium* sp. str. RD4.1 [36], and *Marinosulfonomonas methylotropha* str. TR3 [20] (kind gift of Prof. J. Colin Murrell, University of East Anglia, UK). The extraction of genomic DNA from bacterial strains, previously described or new, was performed with a Maxwell 16 Cell DNA Purification Kit and the Maxwell 16 robot (Promega Corporation), accordingly to the manufacturer’s instructions.

### Seawater sample collection and metagenomic DNA isolation

Atlantic Ocean surface water was collected along the coast of Leça da Palmeira, Portugal (coordinates: 41.226956, -8.720528). Briefly, approximately 8 liters of seawater were collected off the rocky shore at high tide into clean bottles, which were immediately transported to the lab in an isothermal bag with ice packs. Five liters of water were successively filtered through 8 μm, 0.45 μm and 0.2 μm filters. Metagenomic DNA was extracted from the three filters with the PowerWater DNA Isolation Kit (MO BIO Laboratories, Inc.) according to the manufacturer’s instructions.

### Amplification and sequencing of the *msmA* and *msmE* genes

MsmA or MsmE homologs available in the databases were aligned using ClustalW [37] and the corresponding gene sequences were aligned using the output from ClustalW as a scaffold in RevTrans 1.4 [38]. Since the *msmA* and *msmE* genes of *Me*. *methylovora* str. M2 and *Ma*. *methylotropha* str. TR3 are fairly divergent from their Sargasso Sea Metagenome homologs (GenBank accession numbers: EF103447 and EF103448), it proved impossible to design PCR primer pairs common to both types. As such, two primer sets (synthesized by Stabvida Lda., Caparica, Portugal) were employed for each gene: one based on the *Me*. *methylovora* str. M2 sequence and the other based on the SSM sequences. The primer pairs used are listed in S1 Table. The DNA extracted from the coastal seawater sample microbes trapped on 0.2, 0.45 and 8 μm filters was tested both with the *msmA-* and msm*E*-directed primers. Different brands of DNA polymerase were used and several reaction parameters had to be adjusted in order to optimize amplification including the usage of additives betaine and DMSO (S2 Table). We initially used Taq Plus DNA polymerase (Citomed, Portugal), commonly employed in our lab. With the amplification of *msmA* from *Hyphomicrobium* sp. str. P2, however, we only obtained results employing the iProof High-Fidelity DNA Polymerase (Bio-Rad). We used GoTaq G2 Flexi DNA polymerase (Promega Corporation) for the rest of our PCR reactions. In the amplification of *msmE* from seawater metagenomic DNA, despite all the attempts, only tenuous bands were obtained, so a nested PCR approach with primer sets SarE133fwd/SarE1119rev and internal primers SarE322fwd/SarE828rev was carried out. Negative controls received PCR water instead of DNA. Positive controls contained DNA from *Me*. *methylovora* str. M2 or from SSM clone EF103447, accordingly. The sizes of the resulting PCR products were confirmed by gel electrophoresis. Products were then purified from agarose (GRS PCR & Gel Band Purification Kit, GRISP, Portugal) and cloned into *Escherichia coli* str. DH5α competent cells using the pGEM-T Easy vector System (Promega Corporation) followed by sequencing with BigDye Terminator 3 (Applied Biosystems) in an ABI 3730 XL sequencer (Stabvida Lda., Caparica, Portugal) and vector-based primers M13fwd and M13rev. An outline of results and conditions is shown in S2 Table and a full list of the sequences used in this work is presented in S3 Table.

### Genome sequencing of the *Filomicrobium* isolates

The genomes of the two *Filomicrobium* sp. Y and W isolates were sequenced using the MiSeq Illumina sequencing platform by Molecular Research LP (Shallowater, Texas, USA). Coverage was 381x and 294x, respectively. Sequence reads were assembled using NGEN assembler (DNASTAR, Inc.).

### Bioinformatic analysis of the genomic sequencing data

Through local tblastn (http://www.ncbi.nlm.nih.gov/BLAST/) [39] searches, the genes encoding the putative Msm proteins were found in both str. Y and str. W genome sequences. As the 20,300 bp genome region containing the proposed *msm* genes in the two strains was 100% identical, we proceeded with the analysis of the segment from just strain Y (deposited in GenBank under accession number KM879220). For open reading frame (ORF) discovery and annotation we used the Glimmer gene prediction software v3.02 (http://www.ncbi.nlm.nih.gov/genomes/MICROBES/glimmer_3.cgi) [40] as well as the ORF Finder (www.ncbi.nlm.nih.gov/projects/gorf/) [41] program both available on the NCBI platform. Blastp searches were performed in order to support the results.

MsmA and MsmE trees were obtained by subjecting sequence alignments to tree inference by PhyML (Maximum Likelihood method) with 100 bootstrap iterations at the Mobyle site (mobyle.pasteur.fr) [42].

## Results

### Novel *msm* gene sequences obtained from isolated strains and seawater sample DNA.

#### Amplification of msmA sequences

PCR using primers aimed at the *msmA* sequence from *Me*. *methylovora* str. M2 (M2A136fwd and M2A1044rev—S2 Table) was successful with *Filomicrobium* sp. str.s Y and W, *Methylobacterium* sp. str.s P1 and RD4.1, and *Hyphomicrobium* sp. str. P2 and resulted in products around the expected size of 908 bp. With metagenomic seawater DNA, the amplification of a product of the right size (approximately 929 bp) was successfully achieved only with the primer set aimed at the *msmA* genes found in the SSM (SarA124fwd/SarA1053rev), while no amplification was obtained with the primers aimed at soil strain M2. The conditions for successful amplification in each case are summarized in S2 Table.

The sequences of *msmA* from *Filomicrobium* sp. str.s Y and W were 100% identical to each other (these data were later confirmed by the whole genome sequencing of the two strains). Also the sequence from soil *Methylobacterium* sp. str. P1 shared identity and similarity values higher than 99% with that of *Me*. *methylovora* str. M2. In general, the *msmA* gene sequences (and their deduced protein sequences) from the MSA-isolates showed much higher identity to one another (78.7 to 99.3% at the nucleotide level; 84.4 to 99.3% at the amino acid level) than to the SSM sequences (59.4 to 63.4% at the nucleotide level; 73.1 to 75.1% at the amino acid level).

On the other hand, all the *msmA* sequences obtained from seawater metagenomic DNA, here designated as SCA1 to SCA10, revealed higher identity values relatively to the SSM sequences (77.5 to 99.0% at the nucleotide level; 88.2 to 100% at the amino acid level) than to those from the isolated strains (58.9 to 63.3% at the nucleotide level; 70.0 to 75.1% at the amino acid level). This finding is not unexpected, since these latter amplicons were obtained employing primers aimed at the SSM sequences and is in line with the differences in GC% observed: indeed, the *msmA* genes of all strains isolated on MSA have moderately high GC% (55 to 62%) while the SSM *msmA* and the SCA sequences have much lower GC% (36 to 38%) (S3 Table). Sequences SCA1 and SCA3 seemed to form a subset within the seawater group with reciprocal identity values higher than 99%.

#### Amplification of msmE sequences

In the case of *msmE*, primers M2E76fwd and M2E736rev were successfully employed with isolates *Methylobacterium* sp. str. P1 and *Ma*. *methylotropha* str. TR3, resulting in products with approximately 697 bp. The predicted peptide sequences from these two amplicons showed very high similarity values between each other and with *Me*. *methylovora* str. M2 (98.5 to 99.5% amino acid identity and 99.2 to 99.6% identity at the nucleotide level). On the other hand, these sequences were significantly less similar to their SSM homolog (61.9 to 62.8% amino acid identity; 57.8 to 58.3% identity at the nucleotide level). Amplification of the *msmE* gene from the other isolates (*Methylobacterium* sp. str. RD4.1, *Hyphomicrobium* sp. str. P2 and *Filomicrobium* str.s Y and W) failed with both the primer sets aimed at *Me*. *methylovora* str. M2 or aimed at the SSM sequences.

In the case of seawater DNA, amplification with primer set SarE133fwd/SarE1119rev yielded just a tenuous band of the expected size (986bp) and several nonspecific bands: therefore a nested-PCR was employed with a second amplification round using internal primers SarE322fwd/SarE828rev: a band of the expected size (around 500 bp) was obtained, cloned, and 30 clones were inspected by insert analysis. Eighteen clones with inserts of roughly the expected size were sent for sequencing. The 18 sequences obtained were all different and only one corresponded to *msmE* (SCE2). The predicted protein sequence from clone SCE2 was 100% similar (99.33% identity; 95.11% identity at the nucleotide level) to MsmE from SSM clone EF103447 and much more distant from the homologous sequence of *Me*. *methylovora* str. M2, *Methylobacterium* sp. str. P1 or *Ma*. *methylotropha* str. TR3 (72.08 to 72.73% similarity).

In line with what was observed with gene *msmA*, the *msmE* sequences obtained from cultivated strains showed higher GC% contents (49 to 66.3%), while lower values were observed in the metagenomic sequences (39 to 41.5%) (S3 Table).

### Analysis of the genomic region containing *msm* genes from *Filomicrobium* strain Y

**Fig 1:**
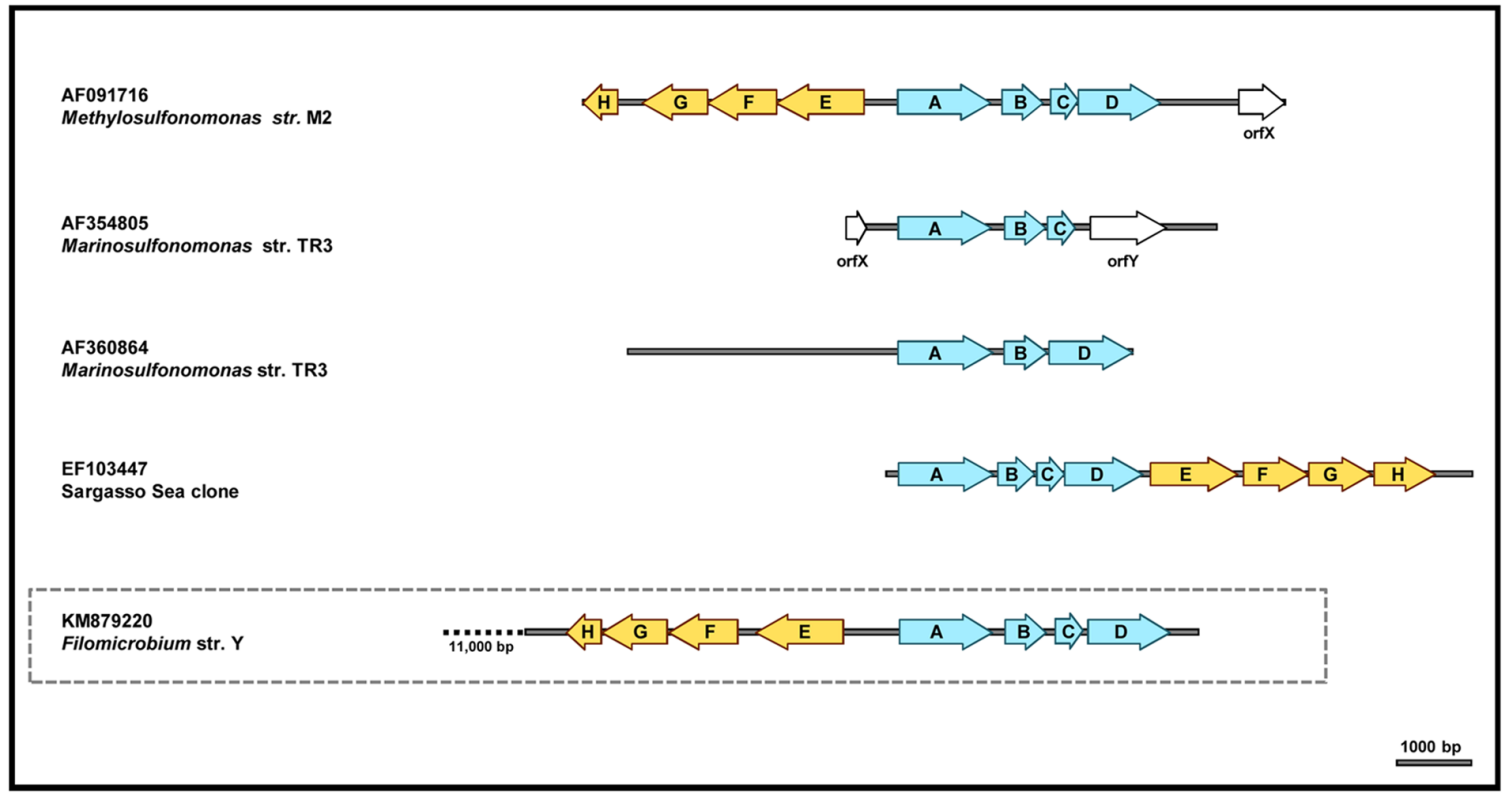
Graphic alignment of the *msm* operons from *Me*. *methylovora* strain M2, *Ma*. *methylotropha* strain TR3, Sargasso Sea clone EF103447 and *Filomicrobium* strain Y. The arrows represent *msm* genes: A, *msmA*; B, *msmB*; C, *msmC*; D, *msmD*; E, *msmE*; F, msmF; G, *msmG*; H, *msmH*. Blue arrows represent genes encoding MSA-monooxygenase and light brown ones correspond to MSA transport genes. *orfX* and *orfY* enconde putative regulators of the *msm* operons [24]. A complete description of all genes on the *Filomicrobium* fragment is provided in Table 1.

Since we were not successful in amplifying the *msmE* gene from *Filomicrobium* sp. strains Y and W, their genomes were sequenced and partially annotated. The two genomes are different but share large regions of identical sequence. A 20,300 bp segment (identical in the two strains; deposited in GenBank under accession number KM879220) was examined with gene prediction bioinformatic tools. The search confirmed the presence of two full *msm* operons, *msmABCD* and *msmEFGH*, and uncovered nine extra open reading frames (see Table 1). The order and arrangement of the *msm* genes within the two operons were very similar to those found in *Me*. *methylovora* str. M2 (see Fig 1).

**Table 1:** Open reading frames annotated on the 20,300 bp genome segment from *Filomicrobium* sp. str. Y.

Hypothetical protein encoded	ORF location	Function
**LysR family transcriptional regulator** ^a^	1417–335	Gene regulation
**Membrane protein sulfite exporter TauE/SafE** ^a^	1688–2449	Sulfite export
**glycine cleavage system protein T** ^a^	2462–3457	Methylotrophic metabolism
**3-methyl-2-oxobutanoate hydroxymethyltransferase** ^a^	3640–4548	C1-moiety transfer
**molybdopterin-binding protein sulfite dehydrogenase (SoxC)** ^a^	4903–6315	Sulfite oxidation
**cytochrome c (SoxD)** ^a^	6389–6967	Sulfite oxidation
**ABC transporter** ^a^	9711–7087	ABC-transport system
**ABC transporter** ^a^	10447–9716	ABC-transport system
**nitrate/sulfonate/bicarbonate ABC transporter ATPase** ^a^	11624–10737	ABC-transport system
**Putative ABC MSA transporter membrane-associated permease component (MsmH)**	12540–11674	MSA transport
**Putative ABC MSA transporter ATP-binding component (MsmG)**	13449–12568	MSA transport
**Putative ABC MSA transporter membrane-associated permease component (MsmF)**	14372–13485	MSA transport
**Putative ABC MSA transporter periplasmic protein (MsmE)**	15705–14569	MSA transport
**MSA monooxygenase, hydroxylase alpha subunit (MsmA)**	16483–17754	MSA metabolism
**MSA monooxygenase, beta subunit (MsmB)**	17941–18432	MSA metabolism
**MSA monooxygenase, ferredoxin (MsmC)**	18546–18923	MSA metabolism
**MSA monooxygenase, reductase (MsmD)**	19010–20056	MSA metabolism

^**a**^ Genes not shown in map of Fig 1.

The two *msm* operons in *Filomicrobium* sp. strains Y and W are divergently transcribed, like in *Me*. *methylovora* str. M2. However, the highest-scoring blast hits for the *msm* genes found on this genomic fragment were obtained with their homologs from marine strain *C*. *Puniceispirillum marinum* str. IMCC1322.

The additional open reading frames found in this genomic fragment downstream of ORF *msmH* (not shown in Fig 1) encode hypothetical proteins associated with ABC-transport systems, with sulfur compounds metabolism (a sulfite exporter and a putative SoxD (cytochrome c)-SoxC (sulfite dehydrogenase) pair) and with reactions linked to methylotrophic pathways (glycine cleavage system protein T or 3-methyl-2-oxobutanoate hydroxymethyltransferase) (see Table 1).

### Conservation of the Rieske-associated motif in the MsmA sequences

All the *msmA* sequences amplified in this work both from MSA-degrading isolates and seawater metagenomic DNA encode a Rieske-associated motif **C**X**H**-X_n_-**C**XX**H** with a conserved 26-amino acid spacer between the two cysteine—histidine groups. This amino acid spacer sequence is much shorter (16 to 18 residues) in non-MSAMO monooxygenases. The longer-than-usual spacer found in MsmA proteins appears to be a constant characteristic in spite of the disparate origins of all the MSA-strains and environmental samples analyzed so far.

### Phylogenetic trees of Msm sequences

**Fig 2:**
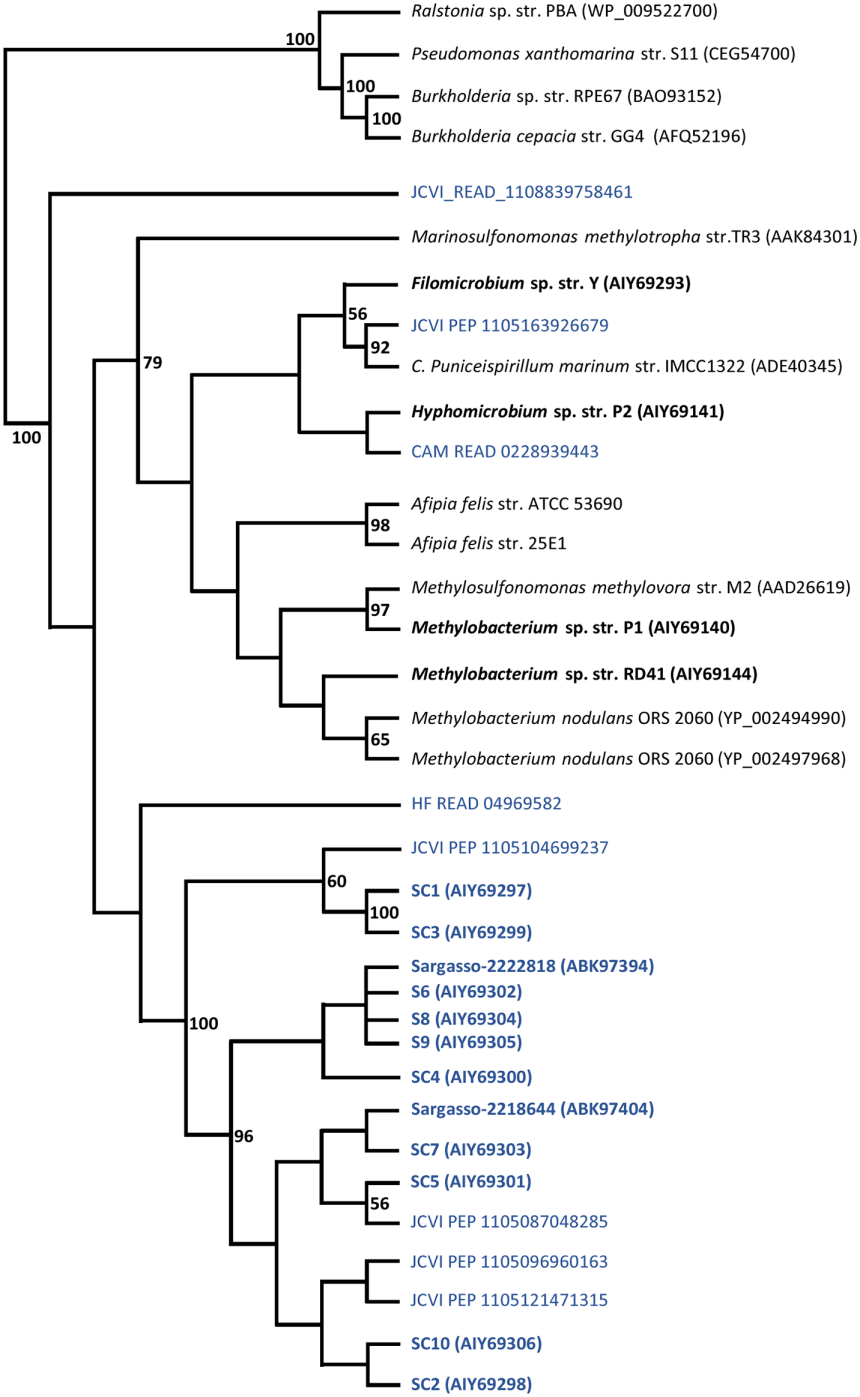
Phylogenetic tree of the MsmA sequences. Novel sequences are in boldface. Marine metagenomic sequences are in blue. Only sequences containing the 26 amino acid spacer in the conserved Rieske-associated motif (**C**X**H**-X_26_-**C**XX**H**) were considered for the analysis. A maximum likelihood method (PhyML) was used for tree inference. Bootstrap values at nodes are for 100 iterations; only values > 50 are shown.

The MsmA sequences obtained in this work were aligned with those previously obtained from cultivated strains, metagenomic SSM sequences EF103447 and EF103448 [15] and similar sequences from the GOS project. The cladogram obtained by PhyML analysis (Fig 2) clearly shows a split into two major groups, one including the *Alphaproteobacteria* and metagenomic data and another corresponding to *Beta* and *Gammaproteobacteria*. Within the *Alphaproteobacteria* branch two subgroups clearly emerge, one comprising all sequences of the cultivated isolates, and the other made up of just seawater metagenomic sequences. Within the group containing *Beta* and *Gammaproteobacteria*, the sequence from *Betaproteobacteria Burkholderia cepacia* GG4 and *Burkholderia sp*. RPE67 curiously show a higher proximity to that of Gammaproteobacterium *Pseudomonas xanthomarina* than to the MsmA from *Ralstonia* PBA (a Betaproteobacterium), which suggests a possible recent event of inter-class horizontal transfer of the *msmA* gene between these strains.

**Fig 3:**
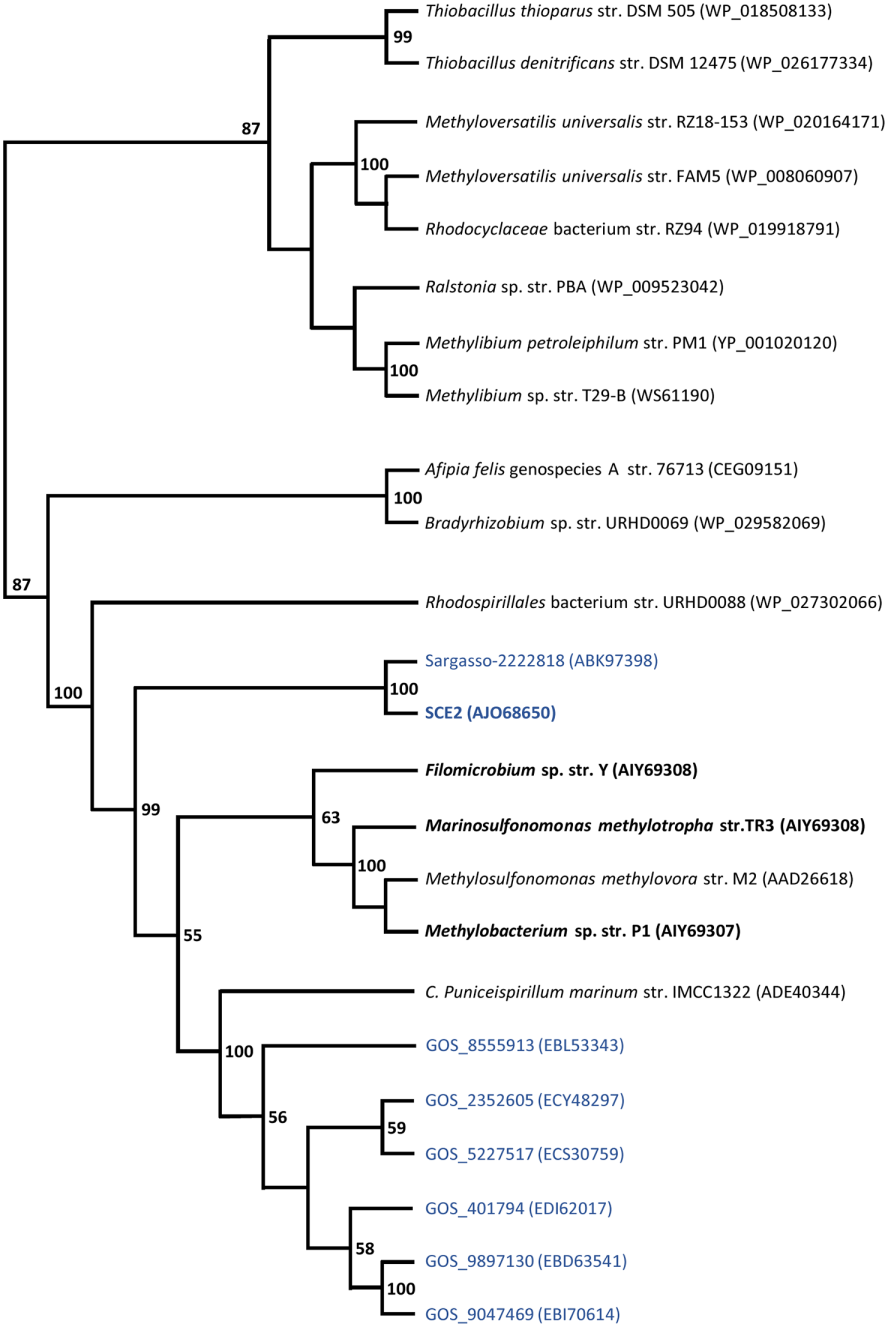
Phylogenetic tree of the MsmE sequences. Novel sequences are in boldface. Marine metagenomic sequences are in blue. A maximum likelihood method (PhyML) was used for tree inference. Bootstrap values at nodes are for 100 iterations; only values > 50 are shown.

The four novel MsmE sequences were aligned with that from *Me*. *methylovora* str. M2 as well as with the highest scoring blastp/tblastn hits of *Me*. *methylovora* str. M2, including SSM EF103448, *C*. *Puniceispirillum marinum* IMCC1322, and GOS sequences. Through the observation of the cladogram (Fig 3) it is easy to recognize two groups of *Alpha* and *Betaproteobacteria*. Within the *Alphaproteobacteria*, a subgroup containing the proteins from low-GC% sequences SCE2 and SSM EF103448 clearly separates from the remaining cluster, which in turn splits in two subgroups, one containing only our novel sequences from cultivated isolates and another including the sequences from GOS and *C*. *Puniceispirillum marinum* IMCC1322.

## Discussion

The quantitative importance of MSA degradation by bacteria in the general biogeochemical sulfur cycle prompts the investigation of the mechanisms underlying MSA catabolism present in these microorganisms.

Methanesulfonate monooxygenase (MSAMO), an enzyme responsible for the first oxidative step of MSA to the central methylotrophic intermediate formaldehyde, was first discovered in soil bacterium *Methylosulfonomonas methylovora* strain M2 and sea strains *Marinoulfonomonas methylotropha* TR3 and PSCH4. After that, the presence of MSA monooxygenase hydroxylase alpha subunit gene (*msmA*) was detected in novel strains able to use MSA as the sole source of carbon and energy.

In the present work PCR was successfully used to amplify *msmA* and *msmE* genes from cultured bacterial strains isolated from different environmental sources (marine surface water, estuarine sediments and soil) and belonging to diverse genera within the *Alphaproteobacteria* class. The amplification of *msmA* from seawater biomass was successful only using a primer pair aimed at sequences previously discovered in the Sargasso Sea Metagenome. Indeed, metagenomic seawater *msmA* and *msmE* sequences have low GC contents and, in general, are phylogenetically close to one another, while the homologs from the cultivated strains showed moderately high GC% and clustered separately. It is well accepted that the culturing of most microorganisms present in environmental samples is not feasible, so it is not surprising that the use of culture-independent methods should yield dissimilar results from traditional laboratory isolation [43]. Indeed, in our results we observe a clear cleavage between culturable MSA-degrading bacteria with moderate to high genomic GC content and most of the uncultured types with low GC%.

All the *msmA* sequences obtained in this work from both isolates and seawater metagenomic DNA were predicted to encode a Rieske-associated motif **C**X**H**-X_26_-**C**XX**H** with a conserved 26-amino acid internal spacer, confirming previous findings [15,18,24,26]. Several predicted proteins annotated as MsmA but completely devoid of the four cysteine/histidine residues (needed to bind the Rieske iron—sulfur cluster) exist in databases (GOS metagenome and virome sequences) [30]. Several other polypeptides with a short spacer (n = 17) have also been described as “MSA monooxygenase large subunits” in methylotrophic strains such as *Methylibium petroleiphilum* str. PM1 [31], *Methyloversatilis universalis* str. FAM5 [32], *Ralstonia* sp. (EIZ03285 and WP_009522700; unpublished), *Rhodocyclaceae* str. RZ94 (WP_019918795; unpublished), and *Thiobacillus* spp. (WP_026177336 and WP_018508129; unpublished). However, we are not aware of any hard data showing that any of these strains do actually employ these polypeptides in the oxidation of MSA and the designation as “MSA monooxygenase” in these cases appears to be merely the consequence of blastp-based automatic annotation. Our results combined with previous findings strongly suggest that the longer-than-usual spacer found in MsmA polypeptides may be a characteristic signature of MSAMOs in spite of the disparate origins of the MSA-utilizing strains and environmental samples analyzed so far. A preliminary structure of the two-compontent hydroxylase of MSAMO has been obtained [44], but specific structure-function studies have not been performed yet so the potential function of such a peculiarly long spacer remains unexplained.

Regarding the MSA/sulfonate transport set of genes (*msmEFGH*), tests were performed with *msmE*, coding for a putative periplasmic substrate binding protein involved in MSA translocation across the membrane [33]. Our attempts were successful only with two of the 6 isolates tested, soil strain *Methylobacterium* P1 and marine strain *Ma*. *methylotropha* TR3. In the case of seawater metagenomic DNA, within the 30 clones analyzed in this work from that amplicon, only one actually contained *msmE* sequence. This greater difficulty we met in amplifying *msmE* relatively to *msmA* is likely due to the lower levels of conservation of the *msmE* gene sequence. This conjecture is also supported by the results one obtains when searching databases using the MsmE sequence as query: much lower numbers of significant hits and lower identity/similarity levels and scores. Consistently, however, the discrepancy in GC% content witnessed with *msmA* sequences was also observed with *msmE*, with higher values in genes obtained from cultivated isolates than directly from seawater DNA (S3 Table).

The *msmE* gene was found to be part of operon *msmEFGH* in *Me*. *methylovora* str. M2 [15,18] and marker exchange mutagenesis data suggested a coordinated expression of this gene with the MSAMO enzyme [33]. This same organization has also been revealed in the whole-genome sequence of *C*. *Puniceispirillum marinum* str. IMCC1322 while in SSM clone EF103447 the two operons are oriented in the same direction. The *Filomicrobium* marine strains Y and W isolated from marine water in this study were shown to contain two full *msm* operons, *msmABCD* and *msmEFGH* adjacent to each other and divergently transcribed (Fig 1). Fittingly, the highest-scoring blast hits for the *msm* genes from these marine isolates were with their homologs from marine strain *C*. *Puniceispirillum marinum* str. IMCC1322. Despite the differences at sequence level, general synteny was demonstrated between the *msm* regions of *Me*. *methylovora* str. M2, *C*. *Puniceispirillum marinum* str. IMCC1322 and *Filomicrobium* strains Y and W.

The annotation of the 20,300 bp segment from the genome of *Filomicrobium* sp. strain Y also yielded nine extra open reading frames localized downstream of ORF *msmH*. Three of these ORFs encoded hypothetical proteins related with sulfite metabolism, namely a sulfite exporter and a SoxD (cytochrome c)-SoxC (sulfite dehydrogenase) pair. According to the literature, periplasmic sulfite dehydrogenases SoxCD (commonly termed “oxidases” in databases) involve a catalytic unit, SoxC, bound to a molybdenum-cofactor (Moco), which feeds electrons into the electron transport chain through a cytochrome c (SoxD) [45,46]. These sulfite dehydrogenases are associated with energy conservation during the oxidation of reduced inorganic sulfur species [46]. Indeed, sulfite dehydrogenases are also present in bacteria degrading longer-chain alkylsulfonates [47,48]. The oxidation of MSA into formaldehyde performed by MSAMO releases sulfite, which can be further oxidized to sulfate either enzymatically or by reaction with oxidizing small molecules. Although this is a process that would allow limited proton-motive force gain, it is theoretically possible to derive energy from the oxidation of the sulfite released from methanesulfonate [10].

In conclusion, this study delivered a palette of 14 novel *msmA* gene sequences (4 from cultivated species and 10 metagenomic) and 4 *msmE* gene sequences (3 from cultivated species and 1 metagenomic). Clearly, the *msmA* genes (and derived proteins) show less sequence variability than *msmE*. This added to the apparently stable conservation of a peculiarly long Rieske-associated motif reinforce the value of gene *msmA* as ecofunctional indicator for methanesulfonate cycling by bacterial natural communities. The data obtained in this work corroborate the suspicion of a strong bias in favor of higher genomic GC-content species when culturing MSA utilizers, especially from marine water. This notion has to be kept in due account in further studies on the degradation of MSA when assessing the representativeness of the results vis-à-vis real natural communities.

## Supporting Information

S1 Table Primers successfully employed in the amplification of *msm*A and *msm*E genes from MSA-degrading isolates and seawater metagenomic DNA. (DOCX)

S2 Table Successful PCR conditions for the amplification of the *msm*A and *msm*E genes from MSA-degrading isolates and seawater metagenomic DNA. In all cases, PCR was performed in 25 μL volume using the manufacturer’s buffer associated with the Taq polymerase employed, 1.5 mM MgSO_4_ and 200 μM of each dNTP.
(DOCX)

S3 Table Listing of amplification and sequencing results ordered by decreasing GC% content. (DOCX)
