# Supplementary material for: Methanesulfonate (MSA) Catabolic Genes from Marine and Estuarine Bacteria
Source: PLoS One. 2015 May 15;10(5):e0125735. doi: 10.1371/journal.pone.0125735 (PMC4433239; doi:10.1371/journal.pone.0125735)
Supplement: S1 Table — (DOCX) [file pone.0125735.s001.docx]

**Table S1.** Primers successfully employed in the amplification of *msmA* and *msmE* genes from MSA-degrading isolates and seawater metagenomic DNA.

|  | **Primer name** | **Sequence (5’→3’)** |
| --- | --- | --- |
| **Primer pairs aimed at *msmA* sequence** | M2A136fwd | CCGTTTCGTCACGAGTCGGAG |
|  | M2A1044rev | ATCTTCATGCAGGTTACGGCCG |
|  | SarA124fwd | AAAAACGTCTGGGTTCCAGTTTGTC |
|  | SarA1053rev | CAAATCTTCATGCAGATTCCTTCC |
| **Primer pairs aimed at *msmE* sequence** | M2E76fwd | CCCGGCGAGAAGGTCGACCTAGTG |
|  | M2E763rev | GAGCGTCGATCTCGGACTCGAGCCAG |
|  | M2E1079rev | ACGCCGATCGGGCTCTGGAGC |
|  | SarE133fwd | TTCGGGAAACCTGGAGAACCTGTAAATTTGG |
|  | SarE322fwd | CATATTGGATACATGGGTGACATGCCC |
|  | SarE828rev | TTGTGCATCTAATTCAGCTTCTAACCAACC |
|  | SarE1119rev | TGGACTCTTTAAGCCACGATCTGC |
