# Supplementary material for: Methanesulfonate (MSA) Catabolic Genes from Marine and Estuarine Bacteria
Source: PLoS One. 2015 May 15;10(5):e0125735. doi: 10.1371/journal.pone.0125735 (PMC4433239; doi:10.1371/journal.pone.0125735)
Supplement: S2 Table — In all cases, PCR was performed in 25 μL volume using the manufacturer’s buffer associated with the Taq polymerase employed, 1.5 mM MgSO4 and 200 μM of each dNTP. (DOCX) [file pone.0125735.s002.docx]

**Table S2.** Successful PCR conditions for the amplification of the *msmA* and *msmE* genes from MSA-degrading isolates and seawater metagenomic DNA.

|  | **Primer pair** | | | | | |
| --- | --- | --- | --- | --- | --- | --- |
|  | **M2A136fwd/M2A1044rev (expected amplicon size: 908 bp)** | | **SarA124fwd/SarA1053rev (expected amplicon size: 929 bp)** | **M2E76fwd/M2E763rev (expected amplicon size: 687 bp)** | | **Nested PCR with primer sets SarE133fwd/SarE1119rev and SarE322fwd/SarE828rev (expected amplicon size: 506 bp)** |
| **Strains** | *Methylobacterium* strain P1, *Filomicrobium* strains Y and W | *Hyphomicrobium* strain P2 | Metagenomic DNA from seawater | *Methylobacterium* strain P1 | *Marinosulfonomonas methylotropha* strain TR3 | Metagenomic DNA from seawater |
| **PCR program** | 94^o^C 5 min + 30 x (94^o^C 1 min + 59^o^C 1 min + 72^o^C 1 min) + 72^o^C 7 min | 98^o^C 30 sec + 30 x (98^o^C 10 sec + 62,8^o^C 30 sec + 72^o^C 30 sec) + 72^o^C 30 sec | 95^o^C 2 min + 35x (94^o^C 1 min + 52^o^C 1 min + 72^o^C 1 min) + 72^o^C 5 min | 94^o^C 5 min + 30 x (94^o^C 1 min + 63,4^o^C 1 min + 72^o^C 1min) + 72^o^C 7 min | 94^o^C 5 min + 30 x (94^o^C 1 min + 69,5^o^C 1 min + 72^o^C 1min) + 72^o^C 7 min | 95^o^C 2 min + 30 x (94^o^C 1 min + (^b^)^o^C 1 min + 72^o^C 1min) + 72^o^C 5 min |
| **Concentrations^a^** | 2 µM forward primer and 0.8 µM reverse primer and 1.5 U of Taq Plus DNA polymerase (Citomed) | 1 µM forward and 0,5 µM reverse primers and 0.5 U of iProof^TM^ High-Fidelity DNA Polymerase (Bio-Rad) | 0.8 µM of forward and reverse primers, 1.25 U of GoTaq® G2 Flexi DNA polymerase (Promega), with 0.125 M betaine and 2.5% DMSO | 2 µM of forward and reverse primers and 2 U of Taq Plus DNA polymerase (Citomed) with 0.125 M betaine and 2.5% DMSO | 2 µM of forward and reverse primers and 2 U of Taq Plus DNA polymerase (Citomed) with 0.125 M betaine and 2.5% DMSO | 0.8 µM of forward and reverse primers, 1.25 U of GoTaq® G2 Flexi DNA polymerase (Promega) |

(^a^) In all cases, PCR was performed in a 25 μL volume using the manufacturer’s buffer associated with the Taq polymerase employed, 1.5 mM MgSO_4_and 200 µM of each dNTP. (^b^) = 54.2^o^C in first PCR and 57^o^C.
