# Supplementary material for: Methanesulfonate (MSA) Catabolic Genes from Marine and Estuarine Bacteria
Source: PLoS One. 2015 May 15;10(5):e0125735. doi: 10.1371/journal.pone.0125735 (PMC4433239; doi:10.1371/journal.pone.0125735)
Supplement: S3 Table — (DOCX) [file pone.0125735.s003.docx]

**Table S3.** Listing of amplification and sequencing results ordered by decreasing GC% content.

| **Gene** | **Designation** | **Gene accession** | **Protein accession** | **Origin** | **GC%^a^** | **Sequence type** |
| --- | --- | --- | --- | --- | --- | --- |
| ***msmA*** | ***Methylobacterium* sp. str. RD41** | KJ789395 | AIY69144 | estuarine sediments | 61.59 | isolate |
|  | *Ralstonia* sp. str. PBA | NZ_AKCV01000022 | WP_009522700 | textile wastewater | 60.53 | isolate |
|  | *Methylobacterium nodulans* ORS 2060 | NC_011892 | YP_002494990 | soil | 60.39 | isolate |
|  | *Methylosulfonomonas methylovora* str*. M2* | AF091716 | AAD26619 | soil | 60.31 | isolate |
|  | *Marinosulfonomonas* *methylotropha* str. TR3 | AF354805 | AAK84301 | marine | 60.24 | isolate |
|  | ***Methylobacterium* sp. str. P1** | KJ789391 | AIY69140 | soil | 60.21 | isolate |
|  | *Afipia felis* str. ATCC 53690 | NZ_KB375270 | WP_002717582.1 | human infection | 57.92 | isolate |
|  | *Afipia felis* str. 25E1 | ABO41866 | ABO41866 | Antarctica lake water | 57.92 | isolate |
|  | *Methylobacterium nodulans* ORS 2060 | EF459501 | YP_002497968 | soil | 56.06 | isolate |
|  | ***Hyphomicrobium* sp. str. P2** | KJ789392 | AIY69141 | soil | 55.94 | isolate |
|  | ***Filomicrobium* sp. str.s Y & W** | KM879220 | AIY69293 | marine | 55.75 | isolate |
|  | *Burkholderia cepacia* str. GG4 | CP003775 | AFQ52196 | soil (rhizosphere) | 55.14 | isolate |
|  | JCVI PEP 1105163926679 ^c^ | JCVI_READ_2101946 | NA | marine | 54.44 | metagenomic |
|  | *Pseudomonas xanthomarina* str. S11 | CCYE01000041 | CEG54700 | soil | 53.88 | metagenomic |
|  | CAM_READ_0228939443 ^c^ | CAM_READ_0228939443 | NA | marine | 53.77 | metagenomic |
|  | *Burkholderia* sp. str. RPE67 | AP014581 | BAO93152 | insect midgut symbiont | 53.04 | isolate |
|  | HF READ 04969582 ^c^ | HF_READ_04969582 | NA | marine | 47.98 | metagenomic |
|  | *C. Puniceispirillum marinum* str. IMCC1322 | CP001751 | ADE40345 | marine | 47.60 | isolate |
|  | JCVI PEP 1105104699237 ^c^ | JCVI_READ_1091140051274 | NA | marine | 47.51 | metagenomic |
|  | JCVI PEP 1112688510920 ^c^ | JCVI_READ_1108839758461 | NA | marine | 42.13 | metagenomic |
|  | JCVI PEP 1105096960163 ^c^ | JCVI_READ_689450 | NA | marine | 38.77 | metagenomic |
|  | JCVI PEP 1105121471315 ^c^ | JCVI_READ_1092214967928 | NA | marine | 37.86 | metagenomic |
|  | Sargasso-2218644 | EF103448 | ABK97404 | marine | 37.82 | metagenomic |
|  | **SCA2** | KP004908 | AIY69298 | marine | 37.68 | metagenomic |
|  | **SCA6** | KP004912 | AIY69302 | marine | 37.68 | metagenomic |
|  | **SCA8** | KP004914 | AIY69304 | marine | 37.57 | metagenomic |
|  | **SCA3** | KP004909 | AIY69299 | marine | 37.46 | metagenomic |
|  | **SCA5** | KP004911 | AIY69301 | marine | 37.46 | metagenomic |
|  | **SCA9** | KP004915 | AIY69305 | marine | 37.46 | metagenomic |
|  | JCVI PEP 1105087048285 ^c^ | JCVI_READ_1066027 | NA | marine | 37.45 | metagenomic |
|  | **SCA4** | KP004910 | AIY69300 | marine | 37.34 | metagenomic |
|  | **SCA7** | KP004913 | AIY69303 | marine | 37.34 | metagenomic |
|  | **SCA1** | KP004907 | AIY69297 | marine | 37.23 | metagenomic |
|  | **SCA10** | KP004916 | AIY69306 | marine | 37.12 | metagenomic |
|  | Sargasso-2222818 | EF103447 | ABK97394 | marine | 36.55 | metagenomic |
| ***msmE*** | *Methylibium* sp. str. T29-B | AZSN01000017 | EWS61190 | freshwater | 66.84 | isolate |
|  | *Methylibium petroleiphilum* str. PM1 | NC_008825 | YP_001020120 | sewage treatment plant biofilter | 66.75 | isolate |
|  | *Methylosulfonomonas methylovora str. M2* | AF091716 | AAD26618 | soil | 66.31 | isolate |
|  | *Rhodospirillales bacterium* str. URHD0088 | NZ_JADL01000017 | WP_027302066 | soil | 65.87 | isolate |
|  | ***Methylobacterium* sp. str. P1** | KP025766 | AIY69307 | soil | 65.36 | isolate |
|  | ***Marinosulfonomonas methylotropha* str. TR3** | KP025767 | AIY69308 | marine | 65.36 | isolate |
|  | *Methyloversatilis universalis* str. RZ18-153 | NZ_ARVV01000001 | WP_020164171 | lake sediments | 63.77 | isolate |
|  | *Rhodocyclaceae* bacterium str. RZ94 | NZ_KB910520 | WP_019918791 | lake sediments | 63.07 | isolate |
|  | *Methyloversatilis universalis* str. FAM5 | NZ_AFHG01000044 | WP_008060907 | lake sediments | 62.89 | isolate |
|  | *Ralstonia* sp. str. PBA | NZ_AKCV01000024 | WP_009523042 | textile wastewater | 58.82 | isolate |
|  | ***Filomicrobium* str.s Y & W** ^b^ | KM879220 | AIY69292 | marine | 56.02 | isolate |
|  | *Thiobacillus denitrificans* str. DSM 12475 | NZ_AQWL01000003 | WP_026177334 | wastewater | 54.84 | isolate |
|  | *Afipia felis* genospecies A str. 76713 | CCAZ020000001 | CEG09151 | hospital water | 54.49 | isolate |
|  | *Bradyrhizobium* sp. str. URHD0069 | NZ_JNIJ01000008 | WP_029582069 | soil | 54.49 | isolate |
|  | GOS_2352605 ^c^ | EP975394 | ECY48297 | marine | 54.29 | metagenomic |
|  | *Thiobacillus thioparus* str. DSM 505 | NZ_KB891326 | WP_018508133 | wastewater | 52.06 |  |
|  | GOS_9047469 ^c^ | EP062324 | EBI70614 | marine | 50.06 | metagenomic |
|  | GOS_9897130 ^c^ | EP340834 | EBD63541 | marine | 49.56 | metagenomic |
|  | *C. Puniceispirillum marinum* str. IMCC1322 | CP001751 | ADE40344 | marine | 48.96 | isolate |
|  | GOS_401794 ^c^ | EP549242 | EDI62017 | marine | 48.51 | metagenomic |
|  | GOS_8555913 ^c^ | EN895414 | EBL53343 | marine | 47.85 | metagenomic |
|  | GOS_5227517 ^c^ | EM024278 | ECS30759 | marine | 46.43 | metagenomic |
|  | **SCE2** | KP167580 | AJO68650 | marine | 41.56 | metagenomic |
|  | Sargasso-2222818 | EF103447 | ABK97398 | marine | 39.18 | metagenomic |

Sequences obtained in this work are shown in boldface. NA = not applicable.

^a^ The GC% values shown here correspond to the maximum length of the *msmA* gene available in each case.

^b^ Obtained by whole genome sequencing.

^c^ GOS metagenome sequences retrieved from the CAMERA database (presently CAMERA Reference Datasets <http://camera.crbs.ucsd.edu/reference-datasets/>). Protein sequences were obtained by virtual translation of metagenomic sequences during tblastn searches.
